# Temperature Dependence of Oxidation Kinetics of Extra Virgin Olive Oil (EVOO) and Shelf-Life Prediction

**DOI:** 10.3390/foods9030295

**Published:** 2020-03-05

**Authors:** Lanfranco Conte, Andrea Milani, Sonia Calligaris, Pierangela Rovellini, Paolo Lucci, Maria Cristina Nicoli

**Affiliations:** 1 Department of Agri-Food, Animal and Environmental Sciences, University of Udine, via Sondrio 2/a, 33100 Udine, Italy; lanfranco.conte@uniud.it (L.C.); milani.andrea.1@spes.uniud.it (A.M.); sonia.calligaris@uniud.it (S.C.); mariacristina.nicoli@uniud.it (M.C.N.); 2 Innovhub Stazioni Sperimentali per l’Industria s.r.l., Via Giuseppe Colombo 79, 20133 Milan, Italy; pierangela.rovellini@mi.camcom.it

**Keywords:** extra virgin olive oil, shelf-life, consumers, oil producers, accelerated shelf-life test, olive oil stability, prediction model

## Abstract

Producers have to guarantee the extra virgin olive oil (EVOO) quality characteristics reported in the Regulation (CEE) 2568/91 throughout the product shelf-life (SL). Unfortunately, due to the development of oxidative reactions, some quality indices change during storage leading to a progressive deterioration of EVOO quality. To avoid the risk of product downgrading in the virgin oil category, the development of effective shelf-life prediction models is extremely important for the olive oil industry. In this research, the accelerated shelf-life testing (ASLT) protocol was applied to evaluate the temperature dependence of selected oxidation indexes as well as to develop a shelf-life predictive model. The evolution of conventional (peroxide value, K232, K270, polyphenols, tocopherols and hexanal) and unconventional parameters (conjugated trienes and pyropheophytin *a*) was monitored in bottled EVOO stored in the dark at increasing temperature (25, 40, 50 and 60 °C). Accordingly, for well-packed products with reduced oxygen in headspace, the best shelf-life index allowing the ability to predict EVOO SL turned out to be K270. In addition, pyropheophytin *a* (%) has been shown to be more sensitive to temperature changes than the secondary oxidation indices, thus suggesting its use as a freshness indicator for storage temperatures higher than 25 °C.

## 1. Introduction

Extra virgin olive oil (EVOO) traditionally represents the major edible vegetable oil consumed in Mediterranean countries. However, today it is globally recognized and appreciated by consumers in non-producing countries. This is due to its unique sensory characteristics combined with the well-demonstrated health-promoting capacity. The latter is mainly associated with a high level of monounsaturated fatty acids (mostly oleic acid) and minor components, such as phenolic compounds and tocopherols [1]. Based on EU Regulation (CEE) 2568/91 as well as International Olive Oil Council (IOC) trade standard [2], the oil extracted from olives by mechanical methods must comply with a number of quality indices to be classified in the extra virgin category [3]. It is a matter of fact that the compliance with these parameters must be guaranteed throughout the product shelf-life to avoid the risk of the product downgrading in the virgin oil category. This situation could be commercially dangerous for producers with possible negative impact on brand reputation. As is well known, many EVOO quality indicators could sharply change during product storage due to the development of oxidative reactions. Typical examples are peroxide number, absorbance values in UV region at 232 and 270 nm and the sensory profile [4]. Beside these compulsory indexes, other indicators, mainly related to oil freshness profile, such as phenol, tocopherol and pigments content, could markedly change during product storage [5,6]. Therefore, the possibility to predict the time needed to exceed the regulatory limits for the EVOO category could be particularly helpful to define the EVOO shelf-life (SL) and thus the “best before” date to be reported on the label based on Regulation EU 1169/2015 [7]. 

As summarized by Nicoli [8], the SL of a packed food product is the length of time after production during which the product retains a specified quality level under well-defined conditions. 

A shelf-life study can be divided into three fundamental steps. The first step implies the identification of the most critical chemical, physical or biological event leading to product quality depletion, followed by the definition of the relevant acceptability limit. Considering EVOO, as previously reported, oxidation is expected to be the main quality deterioration mechanism during storage. Oxidation is usually monitored by a number of indicators, moving from very simple and cheap methodologies to more complex and costly analytical procedures. At present in EU the only available acceptability limits for EVOO are those provided by law (i.e., peroxide value, K232 and K270). Table 1 shows the maximum level of these index allowed by Regulation EC 2568/91. Other early indicators able to monitor product susceptibility to oxidation may also be considered as alternatives or in place of the compulsory ones. However, in this case, the availability of acceptability limits is essential to go forward in a shelf-life study.

In the next step of the SL assessment process, the changes of the selected quality indicators are monitored as a function of time under storage conditions mimicking the foreseeable ones (real-time shelf-life testing) or under environmental conditions able to speed up the deteriorative events (accelerated shelf-life testing—ASLT). Finally, data should be modeled to obtain a shelf-life estimation or prediction, respectively [9,10]. Among all environmental factors potentially applicable to accelerate oxidative reactions, temperature is certainly the most widely used. This is not only due to the fact that temperature is one of the most critical factors affecting food reaction kinetics, but also thanks to the availability of a mathematical description of the temperature sensitivity of quality loss rates, that is the well-known Arrhenius equation, Equation (1) [11]:

(1) $$k=k_{0}\cdot e^{-\frac{E_{a}}{RT}}$$

where *k* is the reaction rate constant; *R* is the molar gas constant (8.31 J K^−1^ mol^−1^), *T* is the absolute temperature (K); *E_a_* is the apparent activation energy (J mol^−1^) and *k*_0_ is the so-called pre-exponential factor. Even if this model has been developed theoretically on the molecular basis for reversible chemical reactions, it has been shown to hold empirically for estimating the rate of a wide range of complex chemical, physical and sensory changes occurring in foods [12,13]. Based on this equation, the temperature sensitivity of deteriorative reactions can be described by the activation energy value (*E_a_*). In the case of lipid oxidation, the range of *E_a_* values moves from about 20 to 150 kJ/mol [10]. This wide range of *E_a_* magnitudes obviously depends on the different food characteristics, processing and storage conditions. For EVOO, Mancebo-Campos et al. [14] reported the temperature dependence from 25 to 60 °C of oxidative reactions by means of the Arrhenius equation, highlighting *E_a_* values around 65 kJ/mol for primary oxidation products and of about 77 kJ/mol for secondary oxidation products. Besides, Calligaris et al. [15] reported, in the same temperature range, *E_a_* values of 42 and 33 kJ/mol for peroxide value and hexanal, respectively. It should be highlighted that in both cases the samples were stored under a high oxygen environment, which is completely different from the actual exposure conditions of EVOO during storage on the market. 

Finally, it should be also stressed that other studies reported the temperature sensitivity of oxidation obtained at elevated temperatures. As stated by Frankel [16] for food lipids, the use of temperatures higher than 60 °C for vegetable oils is questionable, because samples develop excessive rancidity levels, which are not relevant to what happens under normal storage conditions. In agreement with this observation, some accelerated tests developed to evaluate the oxidative stability of oils in a short time (e.g., Rancimat and OSI) showed a low correlation compared with tests conducted under actual storage conditions [10,17]. 

Based on these considerations, the olive oil industry is still in great need of effective shelf-life prediction models based on simple analytical descriptors to be employed for ensuring the product quality during storage on the market. The aim of this paper is to apply the ASLT protocol to evaluate the temperature dependence of different oxidation indexes as well as to develop a shelf-life predictive model. To this purpose, a selected EVOO oil was bottled mimicking commercial conditions and stored at increasing temperature in the dark (25, 40, 50 and 60 °C). During storage conventional (peroxide value, K232, K270, polyphenols, tocopherols and hexanal) and unconventional parameters (conjugated trienes and pyropheophytin *a*) were monitored and relevant oxidation rates were calculated. Rate constants were then modeled to define the temperature dependence, and finally to develop a shelf-life prediction model. 

## 2. Materials and Methods 

### 2.1. Chemicals

Acetone, acetonitrile, isopropanol, ethanol, methanol and *n*-hexane (all HPLC grade) were purchased from Sigma–Aldrich (Milano, Italia). Water was purified with a Milli-Q system (Millipore, Bedford, MA, USA). All other reagents were of analytical grade. Tocopherol (α, γ and δ-tocopherols) and phenolic acids (tyrosol and hydroxytyrosol) standards were purchased from Sigma–Aldrich (Milano, Italia). Chlorophyll A was purchased from Sigma–Aldrich (Milano, Italia).

### 2.2. Olive Oil Samples

Extra virgin olive oil (EVOO) (*Olea europaea* L., cv Coratina) samples were taken from a homogeneous batch produced in 2018. Samples were kindly provided by Castel del Chianti (Tavarnelle Val di Pesa, Firenze, Italy). 

### 2.3. Storage Conditions

Aliquots of 250 mL of EVOO were commercially packed in clear glass bottles with a metal cap and PTFE internal septum. A total of 60 bottles (15 for each selected temperature) were stored at 25, 40, 50 and 60 °C in the dark in incubators (FTC 90I Refrigerated Incubator, Monza, Italy) for up to 300 days. At different storage times, one bottle was taken from the incubators and subjected to analytical determinations. 

### 2.4. Reference Chemical Analysis 

Determination of acidity as oleic acid, peroxide value (PV), specific UV absorption at 232 nm (K232) and 270 nm (K270) and fatty acids methyl esters were conducted according to the procedure reported in Regulation EC 2568/91 and its amendments [2]. Table 1 gives the initial fatty acid composition of EVOO. 

### 2.5. Phenolic Compounds

Phenolic compounds were extracted following the International Oleic Council method [18] and hydrolyzed according to the method proposed by Rovellini et al. [19]. The hydrolyzed sample was then analyzed by UHPLC using an Agilent Poroshell 120 EC-C18 reversed-phase column (2.7 µm particle size, 4.6 × 150 mm) on a Shimadzu Nexera UHPLC System (Shimadzu Nexera, Kyoto, Japan) equipped with dual pump LC-30AD, on-line degasser DGU-20AS, column oven CTO-30A, autosampler SIL-30AC and diode array detector (SPD-M20A). Gradient separation was created from solvent A (water with 2% of acetic acid) and solvent B (acetonitrile) as follows: starting from 95% A; 0.01–12 min linear gradient from 5% to 70% B; 12–13 min linear gradient from 70% to 90% B; isocratic condition kept up to 17 min; 17 min back to initial condition at 5% B; isocratic step kept up to 22 min for column re-conditioning. The mobile phase flow rate was 450 μL min^−1^. The column temperature was 30 °C. Injected volumes for each sample was 5 μL. The detector was set at 280 nm. Polyphenols quantification was obtained using calibration curves obtained by injection on the column of different amounts of both tyrosol and hydroxytyrosol (10–600 ng) with *R*^2^ values higher than 0.999, in all cases.

### 2.6. Tocopherols

UHPLC analysis was realized using a Shimadzu Nexera (Shimadzu, Kyoto, Japan) coupled with the same components used for polyphenols analysis and a fluorescence detector RF-20Axs with double acquisition channels and a 12 µL cell. The detector was set at 296 nm and 325 nm for exciting and emission wavelengths, respectively. Oil samples were diluted in 2-propanol for reaching a 100 mg/mL concentration and 1μL injected on the column as a compromise between sensibility and column capacity.

The chromatographic separation was performed using an Agilent Eclipse PAH column (1.8 µm particle size, 4.6 × 50 mm) under isocratic conditions with solvent A (methanol) and B (acetonitrile) in the ratio 60/40 (*v/v*) and a total flow of 600 μL min^−1^. The oven temperature was set to 30 °C. The injected volume for each sample was 1 μL. 

Tocopherols were quantified using a calibration curve for α, β+γ and δ respectively in the range 0.05–100 ng on the column with *R*^2^ values higher than 0.999.

### 2.7. Pyropheophytin a (ISO 29841:2009 (E))

Pyropheophytin *a* was measured using method ISO 29841:2009 [20]. To isolate pigments was used an SPE SiOH column 6 mL/1 g (Chromabond Macherey-Nagel GmbH & Co, Düren, Germany) using the first 10mL of a petroleum ether/ethyl ether solution in the ratio 90:10 for the elution of non-polar compounds than 10 mL of acetone as elution solvent for chlorophylls fraction. The eluate was then analyzed by reverse-phase Spherisorb ODS2 C18 -HPLC and the separated components were monitored at 410 nm using a photometric detector. The results were expressed as relative proportions (pyropheophytin *a*, %PPP) of the analyses (pyropheophytin *a* and pheophytin *a* and *a’*), in relation to the sum of pyropheophytin *a* and pheophytin *a*+*a*’.

### 2.8. Conjugated Trienes

The analysis of conjugated trienes (CT) was performed following the method proposed by Rovellini and Cortesi [21]. Briefly, the sample was diluted in 1 mL of isopropanol and then analyzed in HPLC/DAD instrument (Finnigan P4000 HPLC, Saint Hose, CA USA), injecting 20 µL. Conjugated benzylester fatty acids derivatives were separated using a RP18 Spherisorb ODS2 column (5 µm particle size, 4 × 250 mm) using a binary solvent system of water and acetonitrile, starting with a 50% of solvent B increased to 100% in the first 50 min, maintained for 15 min and then reverted to 50% at a flow rate of 1000 μL min^−1^. Chromatograms were recorded at 255 nm and a scan spectra acquisition from 200 to 400 nm is necessary to identify al peaks.

### 2.9. Volatile Compound Analysis

Volatile compounds considered in this work are hexanal, octane, nonanal, heptadienal and decadienal that were quantified following the method proposed by Vichi [22].

As an internal standard, a solution of 4-methyl-2-pentanol dissolved in refined oil at the concentration of 45 μg/g was used (Sigma Aldrich, St. Louis, MO, USA). Extraction was performed through an SPME fiber with DVB-Carboxen_PDMS 50/30 μm phase 2 cm long (Agilent Technologies, Santa Clara, CA, USA). The samples were prepared by weighing 2.0 g of sample and adding 50 μL of an internal standard solution in a 10 mL vial closed with a metallic screw cap and PTFE/silicon septum (Agilent Technologies). The sample was equilibrated for 20 min at 40 °C. After the equilibration, a DVB-Carboxen_PDMS 50/30 μm phase 2 cm long SPME fiber (Agilent Technologies, Santa Clara, CA, USA) was exposed to the head-space (HS) for 30 min at 40 °C. Chromatographic analysis was then performed using a GCMS 5977A Extractor Source (Agilent Technologies, Santa Clara, CA), with autosampler CTC for SPME injection with a VF-WAX column (30 m × 0.25 mm I.D. × 0.25 µm) (Agilent Technologies). The analytes were desorbed to the hot injection port of GC for 2 min at 250 °C in a splitless mode. Oven temperature is kept at 40 °C for 10 min, then increased 3 °C/min up to 200 °C and kept for 2 min. Helium was used as a carrier gas at a constant flow of 1 mL/min. Temperatures of transfer line, ionic source and quadrupole were 280, 175 and 150 °C, respectively. Ionization energy was fixed at 70 eV with an acquisition range of 40–350 m/z. Integration and identification were carried out using Agilent Mass Hunter Qualitative Analysis B.06.00 software (Agilent Technologies) with a deconvolution algorithm and NIST 14 library and linear retention indexes.

### 2.10. Kinetics Data Analysis

Apparent zero-order rate constants (*k*) of oxidation indexes as a function of storage time were calculated by linear regression. No lag phase was detected. Only the increasing part of the curves was considered. 

The effect of temperature on the rate of lipid oxidation was evaluated by means of the Arrhenius equation. To make a better estimation of the apparent activation energy a one-step non-linear regression was applied to all data by using the reparametrized Arrhenius equation, in which it was inserted a reference temperature chosen in the middle of the temperature range considered in the experimental plan, Equation (2)

(2) $$\ln k=\ln k_{ref}-\;\frac{E_{a}}{R}\left(\frac{1}{T}-\frac{1}{T_{ref}}\right)$$

where *k* is the apparent reaction rate, *R* is the molar gas constant (8.31 J/K/mol), *T* is the absolute temperature (K), and *k_ref_* is the apparent reaction rate at *T_ref_* (45 °C). T_ref_ was chosen as 318 K, which is the central value of the temperature interval considered in the study. *E_a_* and *k_ref_* were determined by linear regression analysis and used to calculate *k*_0_, Equation (3):

(3) $$k_{0}=e^{\left(\ln k_{ref}+\frac{E_{a}}{RT_{ref}}\right)}$$

### 2.11. Statistical Analysis

Data were expressed as the mean and standard deviation of at least two analytical determinations on two replicated samples. Statistical elaboration was performed with R Software (3.2.2 version, R Project for Statistical Computing; The R Foundation for Statistical Computing, Wien, Austria). Bartlett’s test was used to check the homogeneity of variance, one-way ANOVA was carried out and Tukey’s HSD test was used as a post-hoc test to determine statistically significant differences among means (*p* < 0.05). Linear regression analysis was performed using Microsoft Excel 2016 (Microsoft Corp., Redmond, WA, USA). The goodness of fitting was evaluated by using the coefficient of determination (*R*^2^), the standard error (SE) and the *p*-value (*p*). 

## 3. Results

### 3.1. EVOO Initial Chemical Composition 

The chemical characteristics of olive oil studied are presented in Table 1. According to EVOO limits established by IOC for quality indexes and EU regulation [2], our matrix was classified as extra virgin olive oil. EVOO sample was also characterized by a high level of total tocopherols (225.6 mg/kg) and hydroxytyrosol and its derivatives content after acidic hydrolytic procedure (332.9 mg/kg), thus over the minimum content fixed by the European Food Safety Authority for the health claim on “olive oil polyphenols” (Commission Regulation (EU) 432/2012) (250 mg/kg) [23].

### 3.2. Kinetics of Quality Indicators during Storage at Increasing Temperatures

The development of oxidative reactions in EVOO during storage was monitored at 25, 40, 50 and 60 °C by following the changes of the following parameters: peroxide value, K232, K270, polyphenols, tocopherols, conjugated trienes, hexanal and pyropheophytins.

Some of these selected indices did not show any relevant changes during storage even at the highest temperatures. In particular, neither PV nor ultraviolet coefficient K232 demonstrated a significant increase over time (Supplementary Tables S1–S4). This result highlights that, in the experimental condition tested, primary oxidation products did not further develop during storage without reaching the compulsory limits of 20 meqO_2_/Kg and 2.50 absorbency for PV and K232, respectively. As well known, during oxidation hydroperoxides, as intermediate reaction products, could at the same time be formed and decomposed and the behavior of the relevant analytical indexes is due to the concomitant development of both events. When the rate of formation is higher than that of decomposition, an increase in both PV and K232 is expected. On the other hand, when the decomposition rate prevails, the value of both indices decreases. Thus, when no changes of the primary oxidation indicators are observed, two different situations could be hypothesized: the reaction in the observed time did not proceed or the formation and decomposition rate of hydroperoxides are similar. Our results are also consistent with those found by Brenes et al. [24], who observed negligible changes of the PV and K232 throughout the storage period (1 year at 30 °C) of oil bottled in closed amber glass jars with reduced headspace. On the contrary, Mancebo-Campos et al. [14] revealed a progressive increase of PV and K232 during storage at different temperatures, similarly to data shown by Gómez-Alonso et al. [25] and Calligaris et al. [15]. It should be noted, however, that in these studies samples were stored in the dark in open containers or in the ordinary atmosphere and, thus, under a completely different condition compared to that considered in our study, in which the oxygen content in the container can be assumed as the limiting reactant.

**Figure 1:**
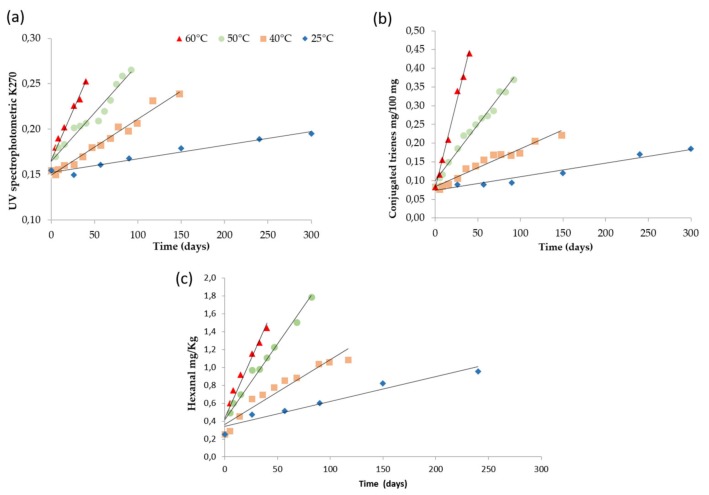
Changes of K270 (**a**), conjugate trienes (**b**) and hexanal (**c**) of extra virgin olive oil stored at 25, 40, 50 and 60 °C (symbols: experimental data, solid line: regression results).

To better understand the evolution of oxidation, the parameter K270 was monitored as an indicator of the secondary oxidation (Figure 1a). A significant increase during the storage of this index was denoted. This result was further confirmed by the increase of conjugated trienes content and hexanal (Figure 1b,c). In fact, k270 which is considered an index of conjugated trienes, could also be influenced by other compounds present in olive oil that could interfere with this measure, such as phenols and other derivatives. On the other hand, the measurement of conjugated trienes by HPLC is specific for the quantification of this class of compounds that derives from the reduction of hydroperoxides of linoleic acid and that can be considered secondary oxidized products. As expected, the rise of the storage temperatures accelerated the changes in these indexes. These findings are in agreement with previous studies [24,26].

**Figure 2:**
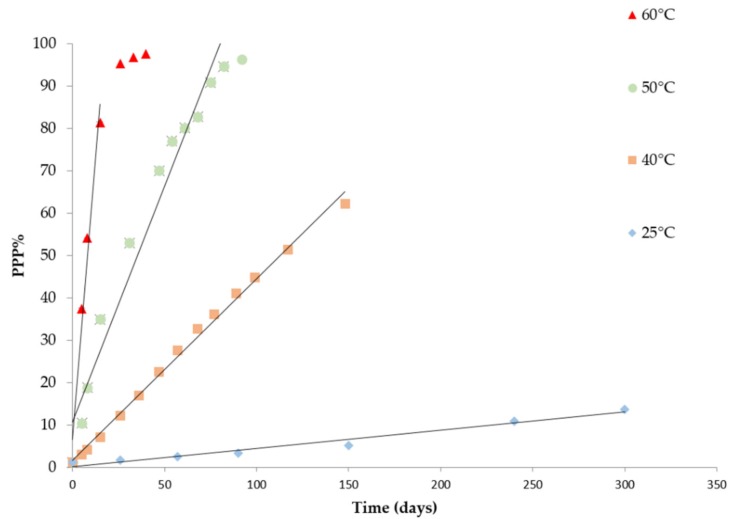
Changes of %PPP of extra virgin olive oil stored at 25, 40 50 and 60 °C (symbols: experimental data, solid line: regression results).

Besides these quality indices, the changes of pyropheophytin *a* during storage was also monitored. Pyropheophytins in olive oil are formed due to degradations of chlorophyll pigments and this reaction begins soon after the oil is extracted. The pigments break down due to a process that involves the decarbomethoxylation of chlorophyll and pheophytins to form pyropheophytins [6]. Figure 2 shows the kinetics of pyropheophytin *a* formation as a function of storage time at 25, 40, 50, and 60 °C. After a first sharp linear increase of this index over time, %PPP reached a plateau at the highest temperatures.

Interestingly, the main antioxidants present in EVOO did not participate in these events as demonstrated by their evolution over time (Supplementary Tables S1–S4). In fact, both total polyphenols and total tocopherols did not show any significant changes upon storage independently on storage temperature.

### 3.3. Modeling the Temperature Dependence of the Oxidation Rate

Based on the results above described, K270, conjugated trienes, hexanal and %PPP resulted in good indicators of product quality depletion during storage at different temperatures. In the attempt to develop a predictive shelf-life model it is fundamental to define the temperature dependence of the rates of these indexes. Considering the data above reported, all the selected indicators followed a pseudo zero reaction order. Thus, the apparent zero-order rate constants were computed by linear regression analysis of the increasing part of the curves reported in Figure 1 and Figure 2. Table 2 shows the values of the rate constants (*k*), the relevant standard error and the coefficient of determination. In all cases, this reaction order well described the evolution of the selected indexes (*R*^2^ > 0.95; *p* < 0.05).

**Figure 3:**
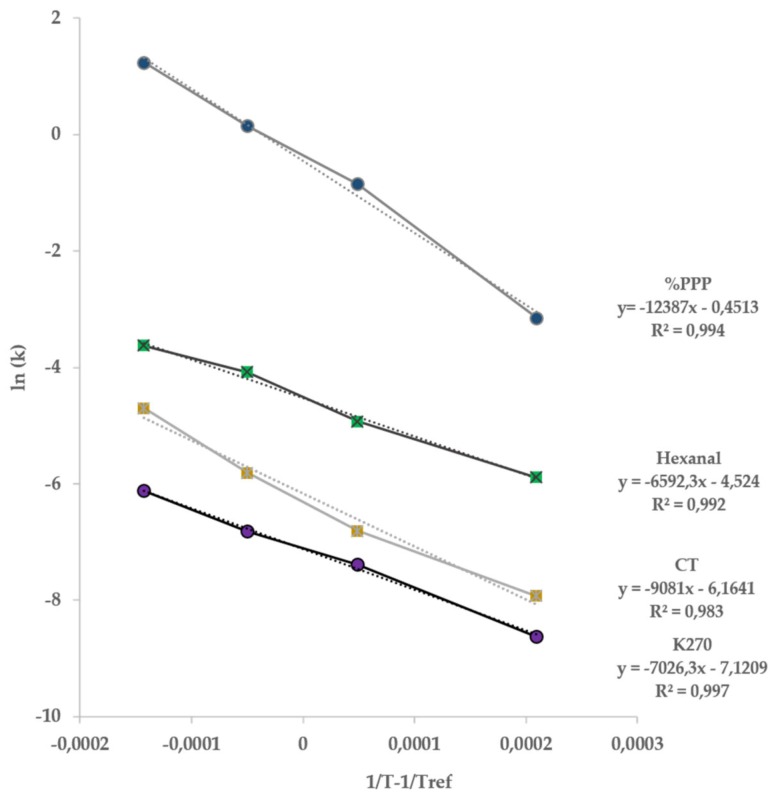
Arrhenius plot of apparent zero-order rate constants of K270, CT and %PPP.

Furthermore, to highlight the temperature dependence of K270, hexanal, conjugated trienes and %PPP, the values of *k* reported in Table 2 were plotted according to the Arrhenius model (Figure 3). In all cases, the Arrhenius behavior was fulfilled in the entire range of temperatures considered (*R*^2^ > 0.97, *p* < 0.05) and *E_a_* and *k*_0_ were calculated by using Equations (2) and (3), respectively (Table 3). It should be remembered in this context, oxidation being a complex reaction, that these values cannot provide a mechanistic interpretation of the reaction, but rather can be used as descriptive tools of the temperature dependence of the reaction [27].

Examining the data in Table 3, it can be noted that the highest values of *E_a_* and *k*_0_ were relevant to the changes of %PPP, highlighting the highest temperature sensitivity of the evolution of this index in comparison to that of the considered secondary oxidation products.

The experimental *E_a_* values acquired were consistent with literature data on lipid oxidation in different matrices and by considering different indices, ranging from 20 to 200 kJ/mol [28]. Considering only references on EVOO, very few information can be found on the application of the ASLT approach. In this regard, an accelerated storage test was applied by Mancebo-Campos et al. [14] to evaluate the temperature dependence of oxidation of EVOO. The authors reported *E_a_* values of about 65 and 76 kJ/mol, respectively for primary and secondary oxidation products. However, it should be noted that these authors stored the oil at increasing temperatures from 25 to 60 °C at a high oxygen concentration. Regarding %PPP temperature dependence, Aparicio-Ruiz et al. [29] studied the thermal degradation of chlorophyll pigments and the consequent formation kinetics of pyropheophytin *a* in virgin olive oil (VOO) in a temperature range from 60 to 120 °C in absence of air. The acquired *E_a_* value was consistent with our research data and around 80–100 kJ/mol. It is well evident the strong impact of the temperature on this index, thus suggesting the use of pyropheophytins as indicator of olive oil quality and freshness, especially if considering that PPP evolution has been proven to be not influenced by the initial quality of the oil, cultivar or growing environments but mainly by storage time, temperature and light exposure [24].

### 3.4. Shelf-Life Estimation

**Figure 4:**
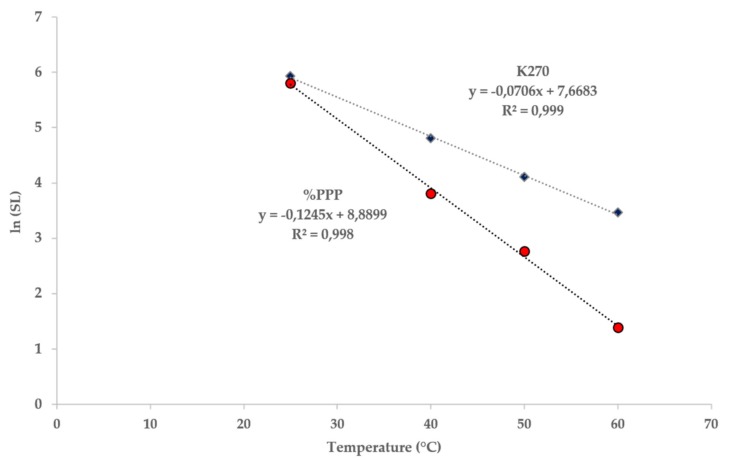
Shelf-life plots of EVOO by using K270 and %PPP as shelf-life indicators.

In the final part of the research, the Arrhenius equations acquired were used as predictive tools to estimate EVOO shelf-life at temperatures below 60 °C. To this aim, as described in the introduction, it is necessary to define a proper acceptability limit [30]. Among the indicators defined by EU regulation, the K270 limit was chosen the only one resulting applicable. This limit, equal to 0.22 (Table 1), represents the threshold value for the EVOO category [2,31]. On the other hand, no compulsory indications in the EU are available for other considered indexes. However, in consideration of the remarkable temperature sensitiveness of the %PPP rate and being considered as freshness indicator by different authors [32,33], the estimation of SL was also carried out by using this indicator. An acceptability limit equal to 17% can be derived from the Australian Standard on EVOO [34]. The following equation was thus used to compute the product SL, Equation (4):

(4) $$\mathrm{SL}=\frac{I_{lim}-I_{0}}{k_{T}}$$

where *I_0_* is the initial value of the selected index, *I_lim_* is the value of the index defined as acceptability limit and *k*_T_ the rate constant at the temperature at which the SL would be defined. This value can be computed by using the Arrhenius equations reported in Figure 4.

By applying Equation (4), SL of EVOO was computed at different temperatures from 25 to 60 °C (Table 4). It is interesting to note that the predicted SL at 25 °C is comparable when using one of the two considered indices. However, as the temperature increased the expected product SL resulted considerably different and, in any case, shorter by considering %PPP above 25 °C. As well known, %PPP has been proposed as a promising freshness indicator because pyropheophytin *a* should be absent or present in trace amounts in freshly prepared EVOO oil [32,33].

The data were further used to generate the so-called shelf-life plot, reporting the ln(SL) as a function of storage temperature. As expected, a good linear relationship between these variables was acquired. The resulting regression lines can be considered the shelf-life predictive models that can be used to estimate EVOO lifetime at any temperature of interest. For instance, the expected SL at 20 °C is 544 and 601 days, considering K270 and %PPP respectively. In this case, K270 represents the early indicator to predict the shelf-life. However, increasing the considered storage temperature, a reverse situation occurs with %PPP becoming a more sensitive indicator. In fact, the SL computed with K270 resulted longer than that predicted by using %PPP (265 days for K270 and 173 days for %PPP).

## 4. Conclusions

In conclusion, results here reported demonstrated the feasibility of the ASLT methodology to develop a predictive tool for shelf-life prediction of EVOO. For well-packed products with reduced oxygen in headspace, we can conclude that nor primary oxidation products neither antioxidant content can be considered as good shelf-life indicators. At the moment, and based on EU regulation, the best SL index allowing to predict EVOO SL resulted K270. This means that by using this simple index and by knowing its temperature dependence, it would be possible to perform an accelerated test at 60 °C able to estimate product shelf-life at ambient temperature in about 1 month.

Particularly interesting resulted also the possible exploitation of %PPP as a freshness indicator. In fact, the changes of pyropheophytin *a* were much more sensitive to temperature changes than the secondary oxidation indices. This parameter could be considered an early indicator of product performances on the market when the expected storage temperature of EVOO was higher than 25 °C.

The evaluation of conjugated fatty acids deriving from the oxidative process, which can be easily evaluated by HPLC, has been also shown to be an interesting new parameter.

From this promising starting point, further research is needed to validate and improve the robustness of the proposed approach by considering oils with different chemical characteristics. In fact, the understanding of the variability of the *E_a_* values and its relationship with EVOO chemical composition could allow generating a general SL model for EVOO.

## Figures and Tables

**Table 1:** Initial composition and characteristics of the studied extra virgin olive oil (EVOO).

Qualitative Characteristics	Values	IOC Reference Values [2,3]
PV (meqO_2_/kg)	5.7	20.0
K232 (ex, 1%, 1cm)	1.81	2.50
K270 (ex, 1%, 1 cm)	0.15	0.22
total tyrosol and hydroxytyrosol (mg/kg)	332.9	n.c.
α-tocopherol (mg/kg)	205.6	n.c.
β+δ-tocopherol (mg/kg)	15.6	n.c.
ϒ-tocopherol (mg/kg)	4.3	n.c.
total tocopherols (mg/kg)	225.6	n.c.
chlorophyllics pigments (mg/kg)	23.5	n.c.
Main Fatty Acids (%)		
C16:0	10.7	7.50–20.00
C18:0	1.9	0.50–5.00
C18:1 ^Δ9c^	76.5	55.00–83.00 *
C18:1 ^Δ11c^	1.2	
C18:2 ^Δ9c,12c^	7.8	2.50–21.00
C18:3 ^Δ9c,12c,15c^	0.6	≤1.00
Others	1.3	---

Legend for fatty acids—*m:n* Δx, *m* = number of carbon atoms, *n* = number of double bonds, x = position of double bonds; n.c.: not considered; * ∑ C18:1^∆9 cis^ + C18:1^∆11 cis^; IOC: International Olive Oil Council. PV: peroxide value.

**Table 2:** Apparent zero-order reaction rate of K270, %PPP and CT of EVOO stored at 25, 40, 50 and 60 °C.

T (°C)	K270		%PPP		CT		Hexanal	
	*k_270_* (D.O.day^−1^·10^−3^)	*R* ^2^	*k_PPP_* (%PPP day^−1^)	*R* ^2^	*k_CT_* (mg/kg day^−1^·10^−3^)	*R* ^2^	*k_hexanal_* (mg/kg day^−1^·10^−3^)	*R* ^2^
25	0.18 ± 0.01	0.96	0.043 ± 0.03	0.99	0.36 ± 0.01	0.99	2.77 ± 0.34	0.95
40	0.62 ± 0.02	0.98	0.428 ± 0.01	0.99	1.05 ± 0.06	0.98	7.24 ± 0.73	0.92
50	1.07 ± 0.07	0.95	1.112 ± 0.07	0.96	3.01 ± 0.11	0.95	16.91 ± 0.94	0.94
60	2.20 ± 0.02	0.97	5.270 ± 0.49	0.97	9.17 ± 0.32	0.96	26.62 ± 2.88	0.97

**Table 3:** Frequency factor (*k*_0_), activation energy (*E_a_*) and corresponding regression parameters of K270, CT, hexanal and %PPP in EVOO.

Index	*k_o_*	*E_a_* (kJ/mol)	*R* ^2^
K_270_	4.88 × 10^12^	58.39	0.99
CT	6.57 × 10^9^	75.46	0.99
Hexanal	9.28 × 10^10^	54.78	0.99
%PPP	1.30 × 10^17^	102.94	0.99

**Table 4:** Estimated shelf-life (days) at 25, 40, 50 and 60 °C by using K270 or %PPP as quality indicators.

Temperature (°C)	Estimated Shelf-Life in Days (months)	
	K270	%PPP
25	377	332
40	122	45
50	61	16
60	32	4

## Supplementary Materials

The following are available online at https://www.mdpi.com/2304-8158/9/3/295/s1, Table S1: Results obtained at 25 °C storage condition for peroxide value, polyphenols, tocopherols and optical density at 232nm at the relative storage times, Table S2: Results obtained at 40 °C storage condition for peroxide value, polyphenols, tocopherols and optical density at 232 nm at the relative storage times, Table S3: Results obtained at 50 °C storage condition for peroxide value, polyphenols, tocopherols and optical density at 232 nm at the relative storage times, Table S4: Results obtained at 60 °C storage condition for peroxide value, polyphenols, tocopherols and optical density at 232 nm at the relative storage times.
